# Detection of ascaridoid nematode parasites in the important marine food-fish *Conger myriaster* (Brevoort) (Anguilliformes: Congridae) from the Zhoushan Fishery, China

**DOI:** 10.1186/s13071-018-2850-4

**Published:** 2018-05-02

**Authors:** Hui-Xia Chen, Lu-Ping Zhang, David I. Gibson, Liang Lü, Zhen Xu, Hai-Tao Li, Hui-Dong Ju, Liang Li

**Affiliations:** 1 Key Laboratory of Animal Physiology, Biochemistry and Molecular Biology of Hebei Province, College of Life Sciences, Hebei Normal University, 050024 Shijiazhuang, Hebei Province People’s Republic of China; 2 Department of Life Sciences, Natural History Museum, Cromwell Road, London, SW7 5BD UK; 3 Medical College of Hebei University of Engineering, 056002 Handan, Hebei Province People’s Republic of China

**Keywords:** Nematode, Anisakidosis, Ascaridoidea, *Conger myriaster*, Zhoushan Fishery, East China Sea

## Abstract

**Background:**

The whitespotted conger *Conger myriaster* (Brevoort) (Anguilliformes: Congridae) is an extremely marketable food fish, commonly consumed as sashimi or sushi in some Asian countries (i.e. Japan, Korea and China). *Conger myriaster* is also suspected as being an extremely important source of human anisakidosis. However, there is currently very little information on the levels of infection with ascaridoid nematode parasites in this economically important marine fish. The aims of the present study are to determine the species composition, prevalence and mean intensity of ascaridoid parasites of *C. myriaster* caught in the Zhoushan Fishery.

**Results:**

A total of 1142 third-stage ascaridoid larvae were isolated from 204 *C. myriaster*. The overall prevalence of infection was 100% (mean intensity 5.6). Nine species of such larvae were accurately identified using integrative taxonomic techniques involving both morphological and genetic data; these included *Anisakis pegreffii*, *A. typica* and *A. simplex* (*sensu stricto*) × *A. pegreffii*, *Hysterothylacium fabri*, *H. aduncum*, *H. sinense*, *H. amoyense*, *H. zhoushanense* and *Raphidascaris lophii*. Although high levels of infection and species richness were revealed in *C. myriaster*, most of the ascaridoid parasites (1135 individuals) were collected from the body cavity and visceral organs of the fish and only seven individuals of *A. pegreffii* were found in the musculature.

**Conclusions:**

This study represents the first report *C. myriaster* from the Zhoushan Fishery being heavily infected with third-stage ascaridoid larvae. Among the ascaridoid larvae parasitic in this fish, an important etiological agent of human anisakidosis, *A. pegreffii* (L3), represents the predominant species. The genus *Hysterothylacium* has the highest species richness, with *H. fabri* (L3) being the most prevalent species. This high level of infection of *A. pegreffii* (L3) in *C. myriaster* suggests a high risk of anisakidosis or associated allergies for people consuming raw or poorly cooked fish originating from this marine area. These findings provide important basic information on the occurrence and infection parameters of ascaridoid nematodes in this economically important marine fish. They also have significant implications for the prevention and control of human anisakidosis when conger eels from the Zhoushan Fishery are consumed.

## Background

Anisakidosis (anisakiasis) is a zoonotic disease well-recognized by the seafood industry [1–5]. Humans become infected by the accidental ingestion of raw or undercooked fish flesh contaminated by ascaridoid larvae, especially anisakids [5–8]. Most cases of human anisakidosis are caused by *Anisakis simplex* (*sensu stricto*), *A. pegreffii* and *Pseudoterranova decipiens*, but species of *Hysterothylacium* and *Contracaecum* have also been implicated [7, 9–12]. During the past three decades, more than 20,000 cases of human anisakidosis have been reported globally, and over 90% of cases are from Japan [6]. Although only one case of human anisakidosis has been reported in mainland China [13], due to the spread of exotic foods (sushi, sashimi, etc.) and the growing consumption of raw or undercooked seafood in mainland China, more attention needs to be paid to this disease.

The whitespotted conger *Conger myriaster* (Brevoort) (Anguilliformes: Congridae) has been considered one of the most common and marketable food-fishes in some Asian countries (i.e. Japan, Korea and China), where it is favoured for consumption raw as sashimi or sushi [14–17]. This fish is mainly distributed between the East China Sea and the waters of Korea and Japan [18]. The Zhoushan Fishery off the coast of China is thought to be the most important fishing ground worldwide for *C. myriaster* [19, 20]. However, there is currently very little information on the levels of infection with ascaridoid parasites in this economically important marine fish. Therefore, the aims of the present study are to determine the species composition, prevalence and mean intensity of ascaridoid parasites in *C. myriaster* caught in the Zhoushan Fishery.

## Methods

### Parasite collection

**Fig. 1:**
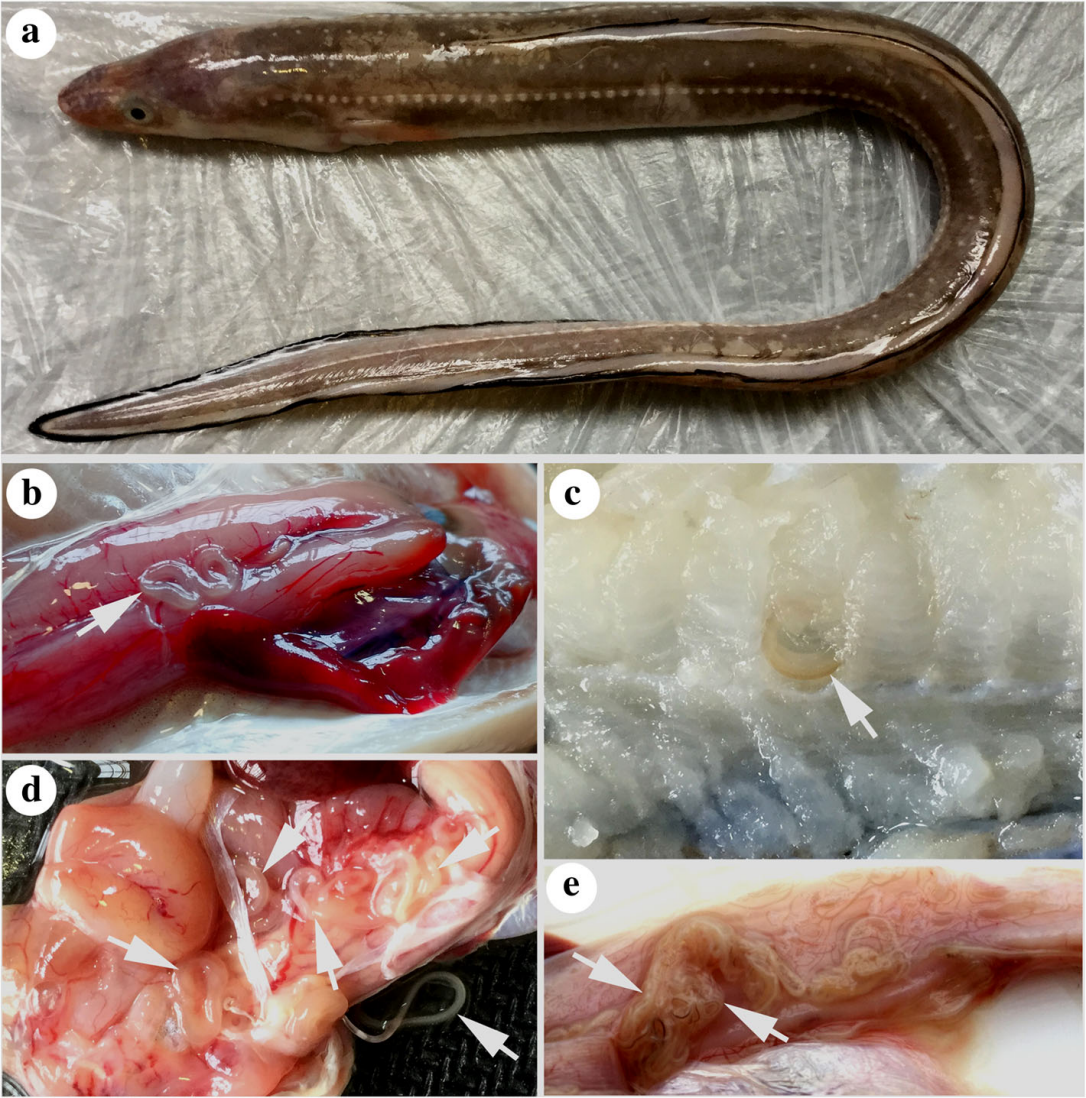
*Conger myriaster* (Brevoort) (Anguilliformes: Congridae) caught in the Zhoushan Fishery, China, heavily infected with ascaridoid nematode parasites. **a** Fish host. **b**, **d**, **e** Large numbers of ascaridoid nematodes present in the visceral organs. **c** Ascaridoid nematode present in the muscles

A total of 204 *Conger myriaster* (Brevoort) (Anguilliformes: Congridae), with a total length (TL) ranging from 25.0–65.0 cm, was dissected, and the body cavity and visceral organs (i.e. digestive tract, mesentery, liver and gonads) were examined for nematode parasites (Fig. 1). The musculature of 54 of these fish (26.5%) (TL 25.0–50.0 cm) was sliced into thin slivers (1.0–2.0 mm thick), and then visually inspected for parasites under white light. All of the fish were caught by commercial trawlers in the Zhoushan Fishery (29°30'–31°00'N, 121°30'–125°00'E) in the South China Sea off China. The nematodes isolated were washed in physiological saline, then fixed and stored in 80% ethanol until studied.

### Morphological identification

The morphology of the nematode larvae was observed using light and scanning electron microscopy. For scanning electron microscopy, specimens were prepared according to the methods used in previous studies [21, 22]. The following morphological characters were used for the identification of different morphotypes of larval nematodes as in previous studies [22–27], including the position of the excretory pore, the absence and presence of an intestinal caecum and a ventricular appendix and their relative lengths, and the morphology of the ventriculus and tail tip.

### Molecular identification

For larval morphotypes with large numbers of individuals, the polymerase chain reaction followed by restriction fragment length polymorphism (PCR-RFLP) analysis and targeted sequencing of the internal transcribed spacer (ITS1-5.8S-ITS2) region of the ribosomal DNA (rDNA) were used for genetic identification. The Column Genomic DNA Isolation Kit (Shanghai Sangon, China) was employed to extract the genomic DNA of each worm according to the manufacturer’s instructions. The ITS1-5.8S-ITS2 region was amplified by PCR using the primers NC5 (5'-GTA GGT GAA CCT GCG GAA GGA TCA T-3') and NC2 (5'-TTA GTT TCT TTT CCT CCG CT-3') [28] under the cycling conditions described previously [25]. PCR products were checked on GoldView-stained 1.5% agarose gel and purified by the Column PCR Product Purification Kit (Shanghai Sangon, China). PCR-RFLP analysis was performed independently using two restriction enzymes *Hinf*I and *Hha*I (Thermo Scientific, Waltham, MA, USA) according to a previous study [29]. The PCR products were digested according to the manufacturer's recommendations. The digested samples were subjected to electrophoresis on 2% agarose gels and then photographed. Representative samples per distinct RFLP profile set were selected for the sequencing of the ITS1-5.8S-ITS2 region. Sequencing was carried out using a DyeDeoxyTerminator Cycle Sequencing Kit (v.2, Applied Biosystems, California, USA) and an automated sequencer (ABI-PRISM 377). Sequencing for each sample was carried out for both strands. Sequences were aligned using ClustalW2 and adjusted manually. The ITS1-5.8S-ITS2 sequences determined were compared (using the algorithm BLASTn) with those available in the National Center for Biotechnology Information (NCBI) database (http://www.ncbi.nlm.nih.gov). For larval morphotypes with small numbers of individuals, the directly targeted sequencing of the ITS1-5.8S-ITS2 region was used for further genetic identification of species with the same primers and methods mentioned above.

### Phylogenetic analysis

Phylogenetic analyses of the ITS1-5.8S-ITS2 sequence data obtained herein were undertaken for both Neighbour-Joining (NJ) and Maximum likelihood (ML) methods using MEGA 6 [30]. We used a built-in function in MEGA 6 [30] to select a best-fitting substitution model for the sequences using the Bayesian information criterion [31]. The Kimura two-parameter model of nucleotide substitution was identified as optimal. *Ascaris lumbricoides* was chosen as the outgroup. Reliabilities for both NJ and ML trees were tested using 1000 bootstrap replications [32] and nodes with bootstrap values exceeding 70 were considered well supported [33].

## Results

### Morphological identification

**Fig. 2:**
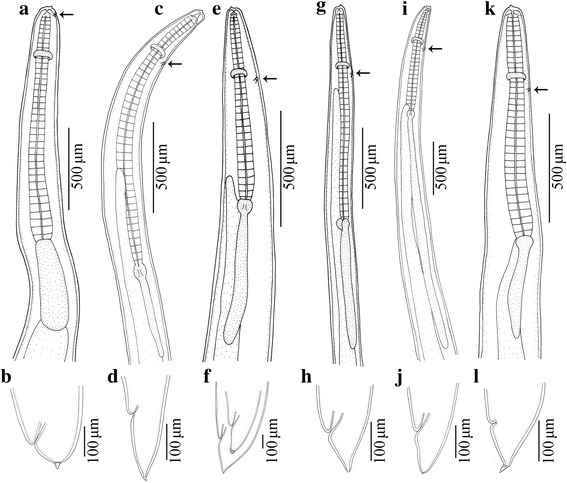
Anterior and posterior extremities of ascaridoid larval morphotypes isolated from *Conger myriaster* (Brevoort) (Anguilliformes: Congridae) caught in the Zhoushan Fishery, China (excretory pore arrowed). **a**, **b,** *Anisakis* type I of Berland (1961) [23]. **c**, **d**, *Hysterothylacium* larval type of Smith (1983) [24]. **e**, **f** *Hysterothylacium* larval type IV of Shamsi et al. (2013) [26]. **g**, **h** *Hysterothylacium* larval type of Guo et al. (2014) [27]. **i**, **j** *Hysterothylacium* larval type of Li et al. (2012) [25]. **k**, **l** *Raphidascaris* larval type of Zhao et al. (2016) [22]

**Fig. 3:**
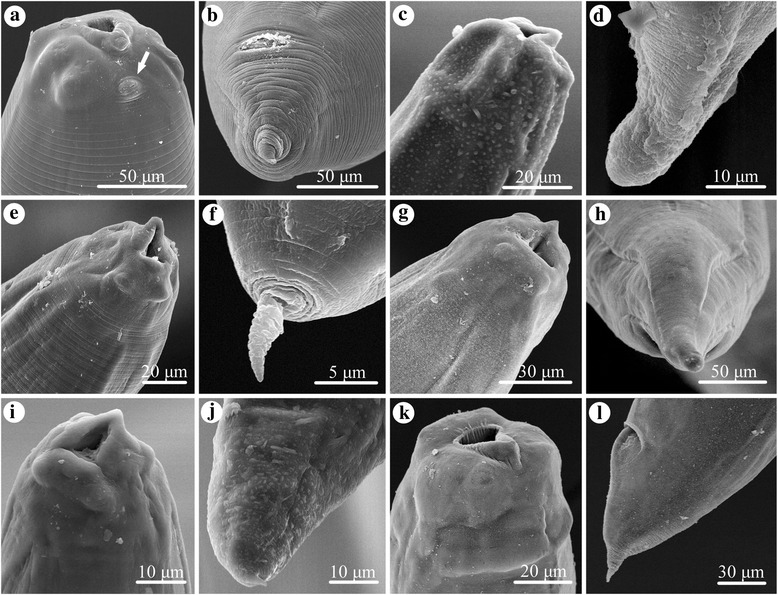
Scanning electron micrographs of the cephalic extremity and tail of ascaridoid larval morphotypes isolated from *Conger myriaster* (Brevoort) (Anguilliformes: Congridae) caught in the Zhoushan Fishery, China. **a**, **b** *Anisakis* type I of Berland (1961) [23] (excretory pore arrowed). **c**, **d** *Hysterothylacium* larval type of Guo et al. (2014) [27]. **e**, **f** *Hysterothylacium* larval type of Smith (1983) [24]. **g**, **h** *Hysterothylacium* larval type of Li et al. (2012) [25]. **i**, **j** *Hysterothylacium* larval type IV of Shamsi et al. (2013) [26]. **k**, **l** *Raphidascaris* larval type of Zhao et al. (2016) [22]

A total of 1142 third-stage ascaridoid larvae were isolated from *C. myriaster*. Based on morphological characters (Figs. 2, 3; Table 1), we found that these larvae represented six different morphotypes belonging to three different genera: *Anisakis*, *Hysterothylacium* and *Raphidascaris*. Among these larvae, 630 individuals were identified morphologically as *Anisakis* type I of Berland (1961) [23]. Four different morphotypes of *Hysterothylacium* spp. larvae were distinguished, including the *Hysterothylacium* larval type of Smith (1983) [24] with 28 individuals, *Hysterothylacium* larval type of Guo et al. (2014) [27] with 23 individuals, *Hysterothylacium* larval type of Li et al. (2012) [25] with 11 individuals and *Hysterothylacium* larval type IV of Shamsi et al. (2013) [26] with 447 individuals. Only three third-stage larvae of *Raphidascaris*, identified morphologically as *Raphidascaris* larval type of Zhao et al. (2016) [22], were found in the present study (see Table 2 for details).

**Table 1:** Morphometric data for ascaridoid larval morphotypes isolated from *Conger myriaster* (Brevoort) (Anguilliformes: Congridae) caught in the Zhoushan Fishery, China (measurements in mm)

	ATB (*n* = 10)	HTS (*n* = 10)	HTIV (*n* = 10)	HTG (*n* = 10)	HTL (*n* = 8)	RTZ (*n* = 3)
BL	9.0–25.0	8.0–21.0	7.0–20.0	9.0–23.0	6.0–19.0	7.0–8.0
OL	0.84–1.93	1.12–1.85	0.62–1.31	1.15–2.18	0.63–1.31	0.66–0.70
VL	0.40–0.79	0.07–0.10	0.05–0.15	0.05–0.10	0.05–0.15	0.05
VW	0.11–0.30	0.06–0.10	0.05–0.14	0.05–0.09	0.05–0.15	0.08–0.09
ICL	–	0.46–0.74	0.13–0.39	0.67–1.29	0.09–0.18	–
VAL	–	0.42–1.27	0.78–2.43	0.70–1.39	1.26–6.29	0.37–0.44
EC	at base of lip	0.35–0.48	0.27–0.47	0.35–0.57	0.28–0.48	0.26–0.27
TL	0.059–0.15	0.14–0.27	0.10–0.27	0.09–0.19	0.12–0.23	0.12–0.13
OL/BL (%)	7.5–13.1	8.8–14.0	6.2–8.9	9.5–13.5	5.2–11.3	8.8–9.4
ICL:VAL ratio	–	1:0.8–2.2	1:3.2–8.1	1:0.9–1.4	1:8.7–48.8	–
ICL/OL (%)	–	24.7–45.2	19.2–37.3	58.1–71.1	10.2–15.8	–

*Abbreviations*: *BL* body length, *OL* oesophagus length, *VL* ventriculus length, *VW* ventriculus width, *ICL* intestinal caecum length, *VAL* ventricular appendix length, *EC* distance from excretory pore to cephalic end, *TL* tail length, *ATB Anisakis* type I of Berland (1961) [23], *HTS Hysterothylacium* larval type of Smith (1983) [24], *HTG Hysterothylacium* larval type of Guo et al. (2014) [27], *HTL Hysterothylacium* larval type of Li et al. (2012) [25], *HTIV Hysterothylacium* larval type IV of Shamsi et al. (2013) [26], *RTZ Raphidascaris* larval type of Zhao et al. (2016) [22]

**Table 2:** Infection data of ascaridoid nematode larvae isolated from *Conger myriaster* (Brevoort) (Anguilliformes: Congridae) caught in the Zhoushan Fishery, China, and samples selected for molecular analysis

Morphotype	Species	Prevalence (%)	Intensity range (mean)	No. of specimens isolated from fish	No. of specimens used for PCR-RFLP analysis	No. of specimens used for sequencing	GenBank ID
ATB	*Anisakis pegreffii*	99.0	1–15 (3.1)	621	621	43^a^	MF539758-MF539767
ATB	*Anisakis pegreffii* × *A. simplex*	3.4	1 (1.0)	7	7	7	MF539768-MF539770; MF539772-MF539774; MF539776
ATB	*Anisakis typica*	1.0	1 (1.0)	2	2	2	MF539771, MF539775
HTIV	*Hysterothylacium fabri*	47.1	1–41 (4.7)	447	447	45^a^	MF539787-MF539796
HTS	*Hysterothylacium aduncum*	11.8	1–5 (1.2)	28	0	28	MF539777-MF539786
HTL	*Hysterothylacium amoyense*	3.4	1–2 (1.2)	7	0	7	MF539807-MF539813
HTL	*Hysterothylacium zhoushanense*	2.0	1 (1.0)	4	0	4	MF539814-MF539817
HTG	*Hysterothylacium sinense*	10.3	1–2 (1.1)	23	0	23	MF539797-MF539806
RTZ	*Raphidascaris lophii*	1.5	1 (1.0)	3	0	3	MF539818-MF539820

^a^Randomly selected

*Abbreviations*: *ATB* *Anisakis* type I of Berland (1961) [23], *HTS* *Hysterothylacium* larval type of Smith (1983) [24], *HTG* *Hysterothylacium* larval type of Guo et al. (2014) [27], *HTL* *Hysterothylacium* larval type of Li et al. (2012) [25], *HTIV* *Hysterothylacium* larval type IV of Shamsi et al. (2013) [26], *RTZ* *Raphidascaris* larval type of Zhao et al. (2016) [22]

### Molecular identification

All the *Anisakis* samples were further identified by PCR-RFLP analysis (Table 2). Digestion of the PCR products using *Hha*I produced two different RFLP profiles, i.e. 628 samples with two bands (*c.*550 and 430 bp) and only two samples with four bands (*c.*320, 240, 180 and 160 bp). Digestion with *Hinf*I yielded three different RFLP profiles, 621 samples with three bands (*c.*370, 330 and 250 bp), only two samples with two bands (*c*.620 and 350 bp) and seven samples with four bands (*c*.620, 370, 300 and 250 bp). According to the molecular taxonomic key based on the PCR-RFLP patterns of *Anisakis* species obtained by the digestion of ITS amplicons with endonucleases *Hha*I or *Hinf*I [29], we assigned these *Anisakis* third-stage larvae to *A. pegreffii*, *A. typica* and *A. simplex* (*sensu stricto*) × *A. pegreffii* (a recombinant genotype). The ITS region was sequenced for 43 randomly selected individuals of *A. pegreffii*, two individuals of *A. typica* and seven individuals of *A. simplex* (*sensu stricto*) × *A. pegreffii* identified by the PCR-RFLP analysis (Table 2). There was no nucleotide variation detected among the 43 ITS sequences of *A. pegreffii* (MF539758-MF539767), two ITS sequences of *A. typica* (MF539771, MF539775) and seven ITS sequences of *A. simplex* (*sensu stricto*) × *A. pegreffii* (MF539768-MF539770, MF539772-MF539774, MF539776). Pairwise comparison between our genetic data and the ITS sequences of *Anisakis* spp. registered in GenBank proved 100% identical to *A. pegreffii* (AY821738, AY821740, AY821745, JQ934867, JQ934871, JQ900763, JQ934869, KP301519, KF032066, KJ011486, EU624343), *A. typica* (EU346093, KC928262, KF356670-KF356671, JX523715, JN968930) and *A. simplex* (*sensu stricto*) × *A. pegreffii* (AB894874).

For the further identification of *Hysterothylacium* spp. third-stage larvae, the ITS region was sequenced for 45 randomly selected individuals of *Hysterothylacium* larval type IV of Shamsi et al. (2013) [26] and all of the individuals of the other three larval morphotypes (Table 2). Four different genotypes (MF539793, MF539794, MF539796, MF539790) were detected among the 45 ITS sequences of *Hysterothylacium* larval type IV of Shamsi et al. (2013) [26] (MF539787-MF539796) obtained herein, which displayed 0–0.2% nucleotide variability. Pairwise comparisons between the present data and the ITS sequences of *Hysterothylacium* spp. registered in GenBank showed 0–0.3% nucleotide differences with *H. fabri* (KC852206, JQ520158, JX974558, KF736939-KF736944). Thus, we considered that the present nematode larvae, referred to as *Hysterothylacium* larval type IV of Shamsi et al. (2013) [26], belong to *H. fabri*. No nucleotide variability was detected among the 23 ITS sequences of *Hysterothylacium* larval type HL of Guo et al. (2014) [27] (MF539797-MF539806). Pairwise comparisons between the present data and the ITS sequences of *Hysterothylacium* spp. registered in GenBank showed them to be 100% identical with *H. sinense* (KX817293-KX817295, KX110078, KX084795). Thus, we confirmed that the nematode larvae referred to as *Hysterothylacium* larval type HL of Guo et al. (2014) [27] belong to *H. sinense*. Only one genotype was detected among the 28 ITS sequences of specimens referred to as *Hysterothylacium* larval type of Smith (1983) [24] (MF539777-MF539786). Pairwise comparisons between the present data and the ITS sequences of *Hysterothylacium* spp. registered in GenBank showed 0–0.5% nucleotide differences with *H. aduncum* (KF736937, HM437225, KP318743-KP318782, KP276150, KT852542). Thus, we considered that the specimens referred to as *Hysterothylacium* larval type of Smith (1983) [24] represent *H. aduncum*. There were seven different genotypes detected among the 11 ITS sequences of *Hysterothylacium* larval type of Li et al. (2012) [25] (MF539807-MF539813, MF539814-MF539817) obtained herein, which displayed 0–1.7% nucleotide variability (Table 3). Pairwise comparisons between the present data and the ITS sequences of *Hysterothylacium* spp. registered in GenBank showed that seven individuals (MF539807-MF539813) exhibited 0–0.3% nucleotide differences with *H. amoyense* (KP252130-KP252133, EU828749), and four individuals (MF539814-MF539817) exhibited 0–1.4% nucleotide differences with *H. zhoushanense* (KP326556, KP326549-KP326551, JX028277-JX028282). We considered, therefore, that the 11 individuals of *Hysterothylacium* larval type of Li et al. (2012) [25] represent two different species, *H. zhoushanense* and *H. amoyense*, with 0.8–1.7% nucleotide variation in the ITS region between these species. Only three third-stage larvae of *Raphidascaris*, identified morphologically as *Raphidascaris* larval type of Zhao et al. (2016) [22], were found. The ITS region was directly sequenced for all of these *Raphidascaris* larvae (Table 2) and the three ITS sequences (MF539818-MF539820) were identical. Comparison of our genetic data with the ITS sequences of *Raphidascaris* spp. registered in GenBank showed them to be 100% identical with *R. lophii* (KP262039, KP326520-KP326531, KP326533-KP326538, KP419720). Consequently, we considered these *Raphidascaris* larvae to be conspecific with *R. lophii.* All of the ITS sequences of the larval nematode parasites obtained herein are deposited in the GenBank database under the accession numbers (MF539758-MF539820).

**Table 3:** Sequence polymorphisms (highlighted in bold) revealed at alignment positions of the ITS region among the different individuals of *Hysterothylacium* larval type of Li et al. (2012) [25] obtained in the present study

	Sequence polymorphisms at alignment positions																	
	96	97	98	220	342	392	685	690	691	696	698	710	807	808	809	812	823	861
*H. amoyense*																		
MF539809	–	–	–	T	T	A	C	A	**A**	A	A	C	–	–	–	C	G	A
MF539811	–	–	–	T	T	A	C	A	C	A	A	C	–	–	–	C	G	A
MF539810	–	–	–	T	T	A	C	A	C	A	A	C	–	–	–	C	G	A
MF539813	–	–	–	T	T	A	C	A	C	A	A	C	–	–	–	C	G	A
MF539808	–	–	–	T	T	A	C	A	C	A	**G**	C	–	–	–	C	G	A
MF539807	–	–	–	T	T	A	C	A	C	A	**G**	C	–	–	–	C	G	A
MF539812	–	–	–	T	T	A	C	A	C	A	**G**	C	–	–	–	C	G	A
*H. zhoushanense*																		
MF539814	**T**	**G**	**G**	T	**A**	**G**	C	**T**	C	**G**	A	**G**	**T**	**T**	**G**	**G**	**T**	**G**
MF539816	–	–	–	**C**	**A**	**G**	C	**T**	C	**G**	A	**G**	**T**	**T**	**G**	**G**	**T**	**G**
MF539817	–	–	–	**C**	**A**	A	**G**	**T**	C	**G**	A	**G**	–	–	–	**G**	**T**	**G**
MF539815	–	–	–	T	T	A	**G**	**T**	C	**G**	A	**G**	–	–	–	**G**	**T**	**G**

### Phylogenetic analysis

**Fig. 4:**
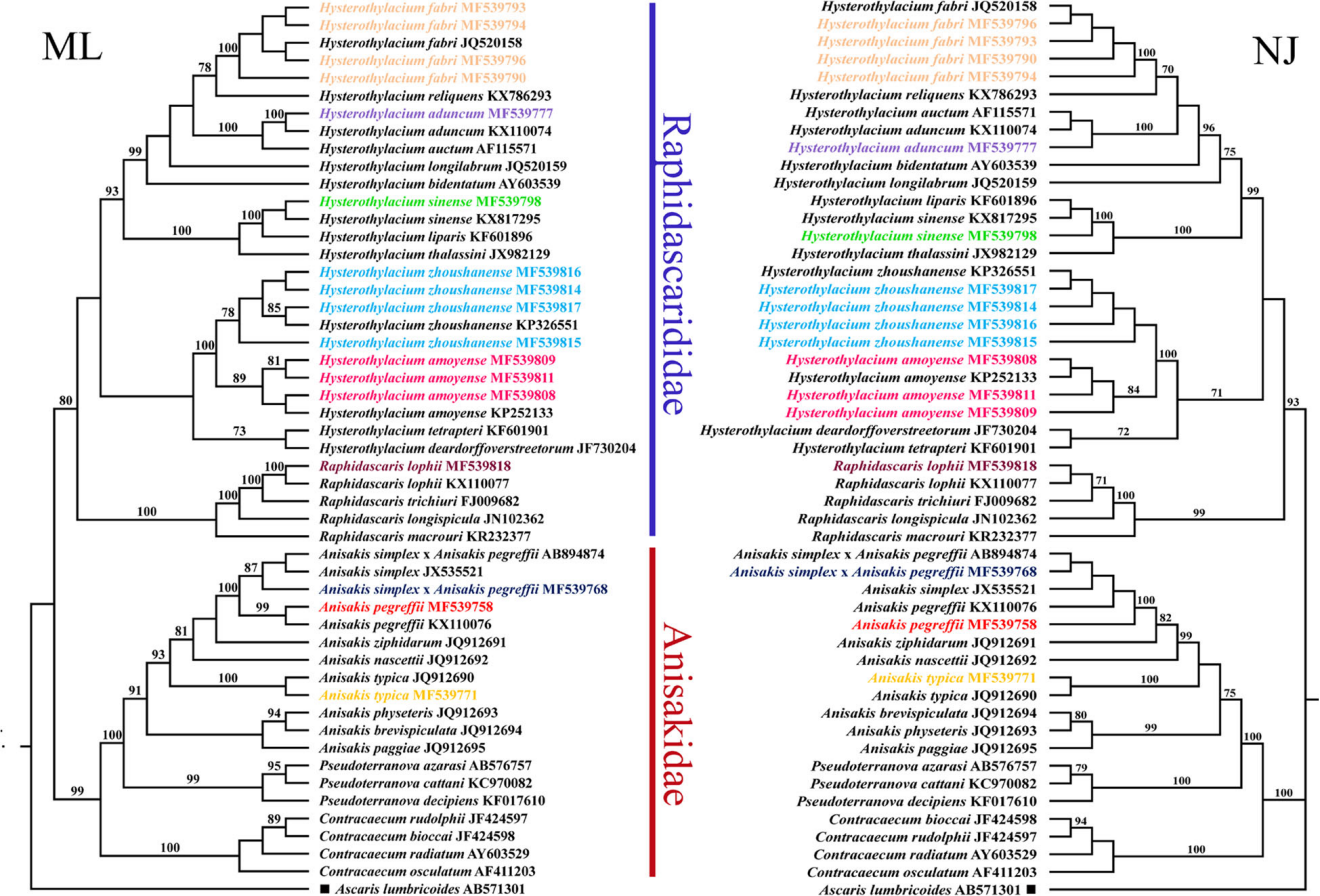
Maximum likelihood (ML) and Neighbour-Joining (NJ) trees showing the phylogenetic relationships of ascaridoid nematode species detected in the present study (shown in colour). *Ascaris lumbricoides* was chosen as the outgroup

Our results revealed that the ascaridoid nematodes selected for phylogenetic analysis were divided into two distinct clades (families), the Anisakidae (which includes species of *Anisakis*, *Pseudoterranova* and *Contracaecum*) and the Raphidascarididae (which includes species of *Hysterothylacium* and *Raphidascaris*) with strong support (Fig. 4). In *Anisakis*, *A. simplex* (JX535521), *A. pegreffii* (MF539758, KX110076), *A. simplex* (*sensu stricto*) × *A. pegreffii* (MF539768, AB894874)*, A. ziphidarum* (JQ912691)*, A. nascettii* (JQ912692) and *A. typica* (MF539771, JQ912690) grouped together, representing *Anisakis* type I of Berland (1961) [23], and *A. brevispiculata* (JQ912694), *A. paggiae* (JQ912695) and *A. physeteris* (JQ912693) formed a group, representing *Anisakis* type II. The results showed that *A. pegreffii*, *A. simplex* and *A. simplex* (*sensu stricto*) × *A. pegreffii* exhibit a very close relationship and that *A. typica* was the basal branch in the *Anisakis* type I group. In *Hysterothylacium*, the four different genotypes of *H. fabri* (MF539793, MF539794, MF539796, MF539790) obtained herein and the previously reported ITS sequence of *H. fabri* (JQ520158) clustered together and as a sister-group to *H. reliquens* (KX786293). *Hysterothylacium sinense* (MF539798, KX817295) formed a sister relationship with *H. liparis* (KF601896). *Hysterothylacium aduncum* (MF539777, KX110074) and *H. auctum* (AF115571) also exhibited a close relationship (Fig. 4). The seven different genotypes of *Hysterothylacium* larval type of Li et al. (2012) [25] obtained herein were divided into two clades: three genotypes (MF539808, MF539809, MF539811) clustered with the previously reported ITS data for *H. amoyense* (KP252133) and four genotypes (MF539814-MF539817) grouped together with a previously reported ITS sequence of *H. zhoushanense* (KP326551) (Fig. 4). *Raphidascaris lophii* (MF539818) was sister to *R. trichiuri* (FJ009682) with high branch support scores (Fig. 4).

### Infection levels

The overall prevalence of infection for ascaridoid larvae in *C. myriaster* was 100% (mean intensity 5.6 worms/fish). *Anisakis pegreffii* (L3) was the predominant species. The prevalence and mean intensity of *A. typica* and *A. simplex* (*sensu stricto*) × *A. pegreffii* was distinctly lower than that of *A. pegreffii*. *Hysterothylacium fabri* (L3) represented the most prevalent species among the *Hysterothylacium* larvae collected from *C. myriaster*. The infection parameters of the other four species of *Hysterothylacium* and *R. lophii* are presented in Table 2. Moreover, in the 54 fish whose muscles were sliced into thin slivers, only seven *Anisakis* larvae, identified genetically as *A. pegreffii,* were found in the musculature, with a prevalence of 2.5% and a mean intensity of 1.0.

## Discussion

*Conger myriaster* represents one of the most valuable fishery resources in some Asian countries (i.e. Japan, Korea and China) [14–17], but it has also been considered an extremely important agent of anisakidosis in Korea [15, 34]. However, our present knowledge of the species composition, prevalence and mean intensity of ascaridoid parasites in *C. myriaster* remains very limited. To date, only one preliminary investigation of the occurrence of ascaridoid larvae in this fish has been undertaken; it was carried out in 1992 and based on specimens purchased from a fish market in Seoul [34]. The study showed that the overall prevalence of infection was 57.7% (of the only 26 fish investigated) [34] and distinctly lower than that (100%) found in *C. myriaster* collected from the Zhoushan Fishery in Chinese waters. However, the mean intensity (90.1) in the previous study is much higher than the value in our investigation (mean intensity only 5.6). The different infection parameters of ascaridoid larvae in *C. myriaster* caught in Korean and Chinese waters may be a result of the different geographical locations or the sampling time (July to August in 1990 *vs* October in 2010 and 2016 and April in 2012 and 2013). In addition, the species composition of ascaridoid larvae detected from *C. myriaster* caught in Korean and Chinese waters is similar. These larval types, i.e. *Anisakis* type I of Berland (1961) [23], *Hysterothylacium* larval type of Smith (1983) [24], *Hysterothylacium* larval type IV of Shamsi et al. (2013) [26] and *Hysterothylacium* larval type of Guo et al. (2014) [27], were found in both our study and the previous one [34], but *Raphidascaris* larval type of Zhao et al. (2016) [22] is only reported in our study. Unfortunately, in the previous study these larvae were not accurately determined to the species level because only morphological methods were used.

The accurate identification of ascaridoid larvae to the species level is essential for an evaluation of the molecular epidemiology of the disease [21, 22, 35]. Recently, the combination of PCR-RFLP analysis and targeted sequencing of the ITS region has been widely used for large-scale studies on the identification of ascaridoid larvae to the species level [11, 29, 35–40]. Consequently, in the present study, in order to accurately identify large numbers of ascaridoid larvae isolated from *C. myriaster* in the Zhoushan Fishery, both morphological methods and molecular approaches, including PCR-RFLP analysis and/or targeted sequencing of the ITS region, were employed, and the six ascaridoid larval types classified herein were identified genetically as nine species.

According to some previous studies [12, 41–45], most cases of human anisakidosis in Europe and Korea are known to be caused by *A. pegreffii* (L3), which is widely distributed in the South Atlantic, the Mediterranean Sea, Australian and Chinese waters (including the Yellow Sea, the East China Sea and the Taiwan Strait) [27, 29, 35, 36, 40, 46–50]. During the present survey, a high level of *A. pegreffii* infection with an overall prevalence of 99.0%, was also revealed in *C. myriaster*, but most of the *A. pegreffii* were found in the body cavity and visceral organs of the fish, which are not eaten by humans, and only seven third-stage larvae of *A. pegreffii* were detected from the musculature of seven *C. myriaster* (prevalence 13.0% and mean intensity 1.0). The relatively low prevalence and intensity of *Anisakis* specimens detected in the musculature of *C. myriaster* suggest a relatively low probability of human infection when consuming *C. myriaster* as sashimi or sushi. However, the post-mortem migration of *Anisakis* larvae from the fish body cavity and/or visceral organs to the musculature can occur after the death of the fish [7, 51, 52], which could increase the risk of anisakidosis when consuming raw or undercooked fish. Consequently, we suggest the removal of the viscera from *C. myriaster* as soon after capture as possible, which would be a useful and practical preventive measure against human anisakidosis [15].

Our genetic data and phylogenetic analysis indicated that the third-stage larvae of *Anisakis* type I of Berland (1961) [23] obtained herein represent three species: *A. pegreffii*, *A. typica* and *A. simplex* (*sensu stricto*) × *A. pegreffii* (Fig. 4). However, based only on morphological characters (i.e. the morphology of the cephalic region, the length of the oesophagus, and the length and morphology of the ventriculus and tail), it is almost impossible to distinguish the third-stage larvae of the three species [37, 46]. The infection levels of *A. typica* (L3) and *A. simplex* (*sensu stricto*) × *A. pegreffii* (L3) are distinctly lower than that of *A. pegreffii* (see Table 2 for details). This situation can be readily understood if one considers the different distributional patterns of these *Anisakis* species in Chinese waters. *Anisakis typica* is mainly distributed in the tropical and subtropical waters [48] and is considered to be the predominant species in the South China Sea [37], but it has also been reported from marine fish species in other Chinese waters (i.e. *Auxis tapeinosoma* and *Chelidonichthys kumu* in the Yellow Sea, *Trichiurus lepturus* in the East China Sea and *Scomber australasicus* in the Taiwan Strait) [29, 40, 46]. The recombinant genotype *A. simplex* (*sensu stricto*) × *A. pegreffii* is found in both temperate and subtropical waters and has frequently been reported from various marine fishes in the Yellow Sea, East China Sea and Taiwan Strait, but all reports indicate low levels of prevalence and intensity [40, 46, 53]. The present study is the first record of *A. simplex* (*sensu stricto*) × *A. pegreffii* from *C. myriaster* in the East China Sea. The significance of *A. typica* and *A. simplex* (*sensu stricto*) × *A. pegreffii* as causative agents of human anisakidosis is distinctly lower than that of *A. pegreffii*. To date, only one case of human anisakidosis caused by the recombinant genotype *A. simplex* (*sensu stricto*) × *A. pegreffii* has been reported; this was from Japan [54]. *Anisakis typica* has not been confirmed as a pathogen causing human anisakidosis.

Species of *Hysterothylacium* are common nematode parasites of marine fishes worldwide [27, 35]. Marine fishes can act as both the paratenic/intermediate and/or the definitive hosts of *Hysterothylacium* spp. [55, 56]. However, most of species of this genus are commonly considered as non-pathogenic to humans. So far, only one case of human anisakidosis apparently caused by *H. aduncum* has been reported [9]. Among the ascaridoid larvae parasitic in *C. myriaster*, *Hysterothylacium* has the highest species richness (five species). Based on the relative length of the intestinal caecum and ventricular appendix, and the morphology of the tail, *Hysterothylacium* larvae can be readily assigned to four different morphotypes (Figs. 2, 3, Table 1). However, it is impractical and problematic to identify these different larval morphotypes to the species level using morphological characters alone [25], thus molecular data were used for the exact identification of species. *Hysterothylacium fabri* (L3) was the most prevalent species among the *Hysterothylacium* spp. larvae obtained herein. The prevalence of *H. fabri* (L3) in *C. myriaster* was similar to that reported for *Liparis tanakae* (Gilbert & Burke) (Scorpaeniformes: Liparidae) collected from the Yellow and East China Seas (prevalence 30.0%) [27]. The present study represents the first record of *H. fabri* (L3) in *C. myriaster*. *Hysterothylacium aduncum* is the most common marine ascaridoid parasite, being reported from more than 220 fish species belonging to 70 families in 22 orders throughout the world [57]. Recently, some authors reported *H. aduncum* (L3) in *L. tanakae* (prevalence of 100%, mean intensity 26.7) and *Pseudorhombus cinnamoneus* (Temminck & Schlegel) (Pleuronectiformes: Paralichthyidae) (prevalence 81.2%, mean intensity 2.7) in Chinese waters [27, 35]. The prevalence and mean intensity of *H. aduncum* (L3) in these two studies were considerably higher than the values in the present study (prevalence 11.8%, mean intensity 1.2). *Hysterothylacium sinense* possibly represents a species endemic to Chinese waters; its type-host is *C. myriaster*. The third-stage larvae of *H. sinense* have been reported from *P. cinnamoneus* in the Yellow Sea off China with a prevalence of 100% and mean intensity of 17.4 [35]. In contrast, we found *H. sinense* (L3) from *C. myriaster* in the East China Sea with a prevalence of only 10.3% and a mean intensity of 1.1. *Hysterothylacium amoyense* (L3) has been reported from several marine fishes, including *Scomber japonicus* and *Trichiurus lepturus* in the East China Sea, *Halieutaea stellata* in the South China Sea [21, 39] and *Platycephalus indicus* (L.) (Scorpaeniformes: Platycephalidae) in the Persian Gulf [58]. In the present study, the prevalence of *H. amoyense* (L3) (3.4%) in *C. myriaster* is distinctly lower than reported in the previous investigation of *Halieutaea stellata* (24.0%) [21]. In the case of the third-stage larvae of *H. zhoushanense,* we found this species in *C. myriaster* for the first time, with a prevalence of 2.0% and mean intensity of 1.0. Previously, it has been reported from *Pseudorhombus oligodon* (Bleeker) (Pleuronectiformes: Paralichthyidae) in the East China Sea, with a prevalence of 5.3% and mean intensity of 7.0 [25].

Our phylogenetic analyses showed that *A. pegreffii*, *A. simplex* (*sensu stricto*) × *A. pegreffii* and *A. simplex* (*sensu stricto*) have much closer relationships than with *A. typica*. Consequently, the present results agree with several previous phylogenetic studies [22, 35, 37, 40, 47, 48]. Some authors have considered that *H. zhoushanense* is closely related to *H. amoyense*, and it is indeed almost impossible to distinguish the third-stage larvae of these two species based on morphological characters [25]. However, adults of *H. zhoushanense,* which have well-developed lateral alae, can be readily differentiated from those of *H. amoyense*, which lack alae. Our phylogenetic analysis corroborated *H. zhoushanense* and *H. amoyense* as separate species and confirmed their sister relationship (Fig. 4).

*Conger myriaster* is bathydemersal and a voracious predator. The high levels of nematode infections and species richness in this fish host are likely related to its feeding habit, which is the main factor affecting parasite community structure [59]. To date, more than 50 species of marine fishes, 40 species of crustaceans, 15 species of molluscs and four species of annelids have been reported in its diet [60]. Most of these prey fishes and invertebrates can act as paratenic or intermediate hosts of the above-mentioned ascaridoid nematode species. We strongly recommended two traditional methods for reducing the risk of human anisakidosis when *C. myriaster* from the Zhoushan Fishery is consumed, i.e. adequate cooking (60 °C for 1–2 min) or freezing (-10 °C for 24 h) of the fish, both of which should kill anisakids [61]. Lastly, the visual inspection of pieces of fish for parasites when preparing sashimi or sushi may be also a good prophylactic measure against human anisakidosis, because *Anisakis* larvae can be detected and eliminated if the pieces of fish are sliced thinly.

## Conclusions

*Conger myriaster* caught in the Zhoushan Fishery was heavily infected with ascaridoid third-stage larvae. Among the ascaridoid larvae parasitic in this fish, the important etiological agent of human anisakidosis, *A. pegreffii* (L3), represented the predominant species. This high infection level of *A. pegreffii* (L3) in *C. myriaster* suggests a high risk of anisakidosis or associated allergies for people consuming raw or poorly cooked raw fish originating from this marine area. The genus *Hysterothylacium* had the highest species richness, and *H. fabri* (L3) was the most prevalent species among the *Hysterothylacium* larvae. The findings of the present study provide important basic data on the occurrence and infection parameters of ascaridoid nematodes in this economically important marine fish. They also have significant implications for the prevention and control of human anisakidosis, which is possible when the flesh of fish caught in the Zhoushan Fishery off the coast of China is consumed raw or inadequately cooked.
